# 64 Higher Levels of Social Participation Are Associated with HRQOL Two Years Post Burn Injury

**DOI:** 10.1093/jbcr/irae036.056

**Published:** 2024-04-17

**Authors:** Emma D Turner, Vanessa Le, Kyra Solis-Beach, Alyssa M Bamer, Kara McMullen, Haig A Yenikomshian, Sarah A Stoycos, Jeffrey C Schneider, Colleen M Ryan, Lewis E Kazis, Caitlin M Orton, Kimberly Roaten

**Affiliations:** University of Texas Southwestern Medical Center, Bedford, Texas; University of Texas Southwestern Medical Center, Grand Prairie, Texas; UT Southwestern Medical Center, Dallas, Texas; University of Washington, Golden, Colorado; University of Washington, Seattle, WA; University of Southern California, Los Angeles, CA; Keck School of Medicine, University of Southern California, Los Angeles, CA; Spaulding Rehabilitation Hospital/Harvard Medical School, Boston, MA; Massachusetts General Hospital/Shriners Children's, Boston, MA; Department of Health Law, Policy and Management, Boston University School of Public Health, Boston, MA; UW Medicine Regional Burn Center, Harborview Medical Center, Seattle, WA; UT Southwestern/Parkland Health, Dallas, Texas; University of Texas Southwestern Medical Center, Bedford, Texas; University of Texas Southwestern Medical Center, Grand Prairie, Texas; UT Southwestern Medical Center, Dallas, Texas; University of Washington, Golden, Colorado; University of Washington, Seattle, WA; University of Southern California, Los Angeles, CA; Keck School of Medicine, University of Southern California, Los Angeles, CA; Spaulding Rehabilitation Hospital/Harvard Medical School, Boston, MA; Massachusetts General Hospital/Shriners Children's, Boston, MA; Department of Health Law, Policy and Management, Boston University School of Public Health, Boston, MA; UW Medicine Regional Burn Center, Harborview Medical Center, Seattle, WA; UT Southwestern/Parkland Health, Dallas, Texas; University of Texas Southwestern Medical Center, Bedford, Texas; University of Texas Southwestern Medical Center, Grand Prairie, Texas; UT Southwestern Medical Center, Dallas, Texas; University of Washington, Golden, Colorado; University of Washington, Seattle, WA; University of Southern California, Los Angeles, CA; Keck School of Medicine, University of Southern California, Los Angeles, CA; Spaulding Rehabilitation Hospital/Harvard Medical School, Boston, MA; Massachusetts General Hospital/Shriners Children's, Boston, MA; Department of Health Law, Policy and Management, Boston University School of Public Health, Boston, MA; UW Medicine Regional Burn Center, Harborview Medical Center, Seattle, WA; UT Southwestern/Parkland Health, Dallas, Texas; University of Texas Southwestern Medical Center, Bedford, Texas; University of Texas Southwestern Medical Center, Grand Prairie, Texas; UT Southwestern Medical Center, Dallas, Texas; University of Washington, Golden, Colorado; University of Washington, Seattle, WA; University of Southern California, Los Angeles, CA; Keck School of Medicine, University of Southern California, Los Angeles, CA; Spaulding Rehabilitation Hospital/Harvard Medical School, Boston, MA; Massachusetts General Hospital/Shriners Children's, Boston, MA; Department of Health Law, Policy and Management, Boston University School of Public Health, Boston, MA; UW Medicine Regional Burn Center, Harborview Medical Center, Seattle, WA; UT Southwestern/Parkland Health, Dallas, Texas; University of Texas Southwestern Medical Center, Bedford, Texas; University of Texas Southwestern Medical Center, Grand Prairie, Texas; UT Southwestern Medical Center, Dallas, Texas; University of Washington, Golden, Colorado; University of Washington, Seattle, WA; University of Southern California, Los Angeles, CA; Keck School of Medicine, University of Southern California, Los Angeles, CA; Spaulding Rehabilitation Hospital/Harvard Medical School, Boston, MA; Massachusetts General Hospital/Shriners Children's, Boston, MA; Department of Health Law, Policy and Management, Boston University School of Public Health, Boston, MA; UW Medicine Regional Burn Center, Harborview Medical Center, Seattle, WA; UT Southwestern/Parkland Health, Dallas, Texas; University of Texas Southwestern Medical Center, Bedford, Texas; University of Texas Southwestern Medical Center, Grand Prairie, Texas; UT Southwestern Medical Center, Dallas, Texas; University of Washington, Golden, Colorado; University of Washington, Seattle, WA; University of Southern California, Los Angeles, CA; Keck School of Medicine, University of Southern California, Los Angeles, CA; Spaulding Rehabilitation Hospital/Harvard Medical School, Boston, MA; Massachusetts General Hospital/Shriners Children's, Boston, MA; Department of Health Law, Policy and Management, Boston University School of Public Health, Boston, MA; UW Medicine Regional Burn Center, Harborview Medical Center, Seattle, WA; UT Southwestern/Parkland Health, Dallas, Texas; University of Texas Southwestern Medical Center, Bedford, Texas; University of Texas Southwestern Medical Center, Grand Prairie, Texas; UT Southwestern Medical Center, Dallas, Texas; University of Washington, Golden, Colorado; University of Washington, Seattle, WA; University of Southern California, Los Angeles, CA; Keck School of Medicine, University of Southern California, Los Angeles, CA; Spaulding Rehabilitation Hospital/Harvard Medical School, Boston, MA; Massachusetts General Hospital/Shriners Children's, Boston, MA; Department of Health Law, Policy and Management, Boston University School of Public Health, Boston, MA; UW Medicine Regional Burn Center, Harborview Medical Center, Seattle, WA; UT Southwestern/Parkland Health, Dallas, Texas; University of Texas Southwestern Medical Center, Bedford, Texas; University of Texas Southwestern Medical Center, Grand Prairie, Texas; UT Southwestern Medical Center, Dallas, Texas; University of Washington, Golden, Colorado; University of Washington, Seattle, WA; University of Southern California, Los Angeles, CA; Keck School of Medicine, University of Southern California, Los Angeles, CA; Spaulding Rehabilitation Hospital/Harvard Medical School, Boston, MA; Massachusetts General Hospital/Shriners Children's, Boston, MA; Department of Health Law, Policy and Management, Boston University School of Public Health, Boston, MA; UW Medicine Regional Burn Center, Harborview Medical Center, Seattle, WA; UT Southwestern/Parkland Health, Dallas, Texas; University of Texas Southwestern Medical Center, Bedford, Texas; University of Texas Southwestern Medical Center, Grand Prairie, Texas; UT Southwestern Medical Center, Dallas, Texas; University of Washington, Golden, Colorado; University of Washington, Seattle, WA; University of Southern California, Los Angeles, CA; Keck School of Medicine, University of Southern California, Los Angeles, CA; Spaulding Rehabilitation Hospital/Harvard Medical School, Boston, MA; Massachusetts General Hospital/Shriners Children's, Boston, MA; Department of Health Law, Policy and Management, Boston University School of Public Health, Boston, MA; UW Medicine Regional Burn Center, Harborview Medical Center, Seattle, WA; UT Southwestern/Parkland Health, Dallas, Texas; University of Texas Southwestern Medical Center, Bedford, Texas; University of Texas Southwestern Medical Center, Grand Prairie, Texas; UT Southwestern Medical Center, Dallas, Texas; University of Washington, Golden, Colorado; University of Washington, Seattle, WA; University of Southern California, Los Angeles, CA; Keck School of Medicine, University of Southern California, Los Angeles, CA; Spaulding Rehabilitation Hospital/Harvard Medical School, Boston, MA; Massachusetts General Hospital/Shriners Children's, Boston, MA; Department of Health Law, Policy and Management, Boston University School of Public Health, Boston, MA; UW Medicine Regional Burn Center, Harborview Medical Center, Seattle, WA; UT Southwestern/Parkland Health, Dallas, Texas; University of Texas Southwestern Medical Center, Bedford, Texas; University of Texas Southwestern Medical Center, Grand Prairie, Texas; UT Southwestern Medical Center, Dallas, Texas; University of Washington, Golden, Colorado; University of Washington, Seattle, WA; University of Southern California, Los Angeles, CA; Keck School of Medicine, University of Southern California, Los Angeles, CA; Spaulding Rehabilitation Hospital/Harvard Medical School, Boston, MA; Massachusetts General Hospital/Shriners Children's, Boston, MA; Department of Health Law, Policy and Management, Boston University School of Public Health, Boston, MA; UW Medicine Regional Burn Center, Harborview Medical Center, Seattle, WA; UT Southwestern/Parkland Health, Dallas, Texas; University of Texas Southwestern Medical Center, Bedford, Texas; University of Texas Southwestern Medical Center, Grand Prairie, Texas; UT Southwestern Medical Center, Dallas, Texas; University of Washington, Golden, Colorado; University of Washington, Seattle, WA; University of Southern California, Los Angeles, CA; Keck School of Medicine, University of Southern California, Los Angeles, CA; Spaulding Rehabilitation Hospital/Harvard Medical School, Boston, MA; Massachusetts General Hospital/Shriners Children's, Boston, MA; Department of Health Law, Policy and Management, Boston University School of Public Health, Boston, MA; UW Medicine Regional Burn Center, Harborview Medical Center, Seattle, WA; UT Southwestern/Parkland Health, Dallas, Texas

## Abstract

**Introduction:**

Burn survivors experience lower levels of health-related quality of life (HRQOL) compared to U.S. population norms even one year post-injury. Research suggests that social support predicts functional outcomes, and while studies comparing life satisfaction pre-burn and at follow-up can begin to elucidate HRQOL following burn injury, there is a paucity of research focusing on the role of social participation in HRQOL. The current study sought to examine the roles of social participation and satisfaction with life pre-injury on HRQOL at two years post-injury.

**Methods:**

Analyses were completed using data from a large national multi-center database. A total of 139 participants enrolled from October 2018 to May 2022 were included (34% female, mean age ± SD = 46.3 ± 16.9, mean TBSA burned ± SD = 19.4% ± 19.7). Measures of social participation included: LIBRE (Life Impact Burn Recovery Evaluation) Social Activities (SA) and Social Interaction (SI) subscales collected at 24-months post-injury. Measure of HRQOL included: VR-12 mental component summary score (MCS) and physical component summary score (PCS) collected at 24-months post-injury. Additional measures included: Satisfaction with Life Scale (SWLS) and Community Integration Questionnaire (CIQ) collected at discharge for pre-injury recall. Four regression models with an interaction term were run to analyze how SWLS pre-injury impacts the relationships amongst LIBRE-SA, LIBRE-SI, VR-12 MCS, and VR-12 PCS while controlling for pre-injury community participation (CIQ).

**Results:**

After adjusting for preinjury CIQ, LIBRE-SI and LIBRE-SA were both positively associated with MCS (p < 0.001). While LIBRE-SA was positively associated with PCS (p < 0.001), LIBRE-SI was not significant. There was no evidence to support an interaction/moderation effect of the relationship between LIBRE-SA or LIBRE-SI and VR-12 MCS or VR-12 PCS by SWLS pre-injury.

**Conclusions:**

Findings suggest higher levels of social participation are associated with increased mental HRQOL. However, only higher levels of social activities were related to increased physical HRQOL. The impact of increased social participation on improvements in both mental and physical HRQOL should be further investigated to inform program development and education for burn survivors on avenues to improve overall HRQOL. Further investigation is warranted to confirm the impact of life satisfaction pre-injury on these results.

**Applicability of Research to Practice:**

These results reinforce the importance of social participation during recovery from burn injury, particularly as it relates to mental health.

